# Catheter-related bloodstream infection due to *Acinetobacter ursingii* in a hemodialysis patient: case report and literature review

**DOI:** 10.11604/pamj.2021.39.208.30565

**Published:** 2021-07-22

**Authors:** Arsanios Martin Daniel, Diana Garzón, Andrés Vivas, Tíĵaro Merchán Viviana, Diego Alejandro Cubides-Diaz, Yesid Mantilla Fabian

**Affiliations:** 1 Department of Internal Medicine, Universidad de la Sabana, Chía, Colombia,; 2 Faculty of Medicine, Universidad de la Sabana, Chía, Colombia,; 3 Faculty of Medicine, Fundación Universitaria Juan N Corpas, Bogotá, Colombia

**Keywords:** Acinetobacter infection, bacteremia, hemodialysis, catheter related infection, case report

## Abstract

Acinetobacter ursingii is an anaerobic gram negative opportunistic coccobacillus, rarely isolated in bacteremic patients. It is mainly found in immunocompromised and severely ill patients with no identifiable source of infection. When isolated into the bloodstream, it usually displays resistance to at least two antimicrobial agents. To date only seven cases of bacteremia due to this microorganism have been reported in adults, of which, this accounts for the second one associated to renal replacement therapy and the first case of a documented catheter-related bloodstream infection (CRBSI) in a patient with a hemodialysis catheter. A 78-year-old male presented into the emergency department with acute kidney injury requiring hemodialysis, later developing bacteremia due to Acinetobacter ursingii.

## Introduction

The genus *Acinetobacter* comprises a group of gram-negative coccobacillus, aerobic, opportunistic, non-fermenter and oxidase-negative bacteria; frequently found in humid environments. *Acinetobacter ursingii* can be found within its genomic species [1]. We present a case of a 78-year-old patient who attended the emergency department for respiratory distress secondary to a blunt chest trauma, with progresive organ dysfunction requiring invasive mechanical ventilation and hemodialysis. After the instauration of renal replacement therapy, the patient presented signs of systemic inflammatory response, with posterior isolation of *A. ursingii* in peripheral blood and catheter-tip cultures.

## Patient and observation

**Patient information:** a 78-year-old male patient, with smoking history for 30 years and arterial hypertension presented in the emergency department due to acute blunt chest trauma.

**Clinical findings:** he was in bad-looking shape, with respiratory distress and altered level of consciousness. Vital signs were as follows: blood pressure 80/62 mmHg, heart rate 120 bpm, respiratory rate 28 bpm, oxygen saturation 70% on room air, and a Glasgow coma scale of 6. At physical examination he presented with generalized paleness, multiple abrasions on the anterior thorax, thoracoabdominal dissociation and bilateral decrease in breath sounds.

**Timeline of current episode:** orotracheal intubation was performed, with a later discovery of a hemopneumothorax requiring closed thoracostomy and red blood cell transfusion. Laboratory findings on admission and during hospitalization are presented in Table 1. The patient presented torpid evolution and multiorgan dysfunction, with hemorrhagic shock, acute liver and renal injury and respiratory distress. Acute physiology and cronic health evaluation (APACHE) and sepsis related organ failure assessment (SOFA) scores were 16 and 11 respectively. At day three of admission renal replacement therapy was initiated under continuous venovenous hemofiltration modality, but five days later the patient presented clinical deterioration with tachycardia, leukocytosis, neutrophilia, lactic acidosis and requirement of increasing doses of vasopressors.

**Table 1:** laboratory findings on admission and during hospitalization

Laboratory	Admission	Day 8	Day 16
White blood cell count (103/μL)	14.05	19.23	12.97
Neutrophils (103/μL)	12.12	15.42	10.10
Hemoglobin (g/dL)	7.6	9.2	9.1
Platelets (103/μL)	60.02	42.58	47.62
Creatinine (mg/dL)	2.5	7.3	4.3
Blood urea nitrogen (mg/dL)	50.2	102	40.6
AST (U/L)	80.3	534	621
ALT (U/L)	74.25	624	701
Total bilirubin (mg/dL)	1.7	2.6	2.7
Indirect bilirubin (mg/dL)	0.68	1.26	1.38
Direct bilirubin (mg/dL)	1.02	1.34	1.32
PaO_2_/FiO_2_ ratio	102	89	70

AST: aspartate aminotransferase; ALT: alanine aminotransferase; PaO_2_: partial pressure of oxygen; FiO_2_: fraction of inspired oxygen

**Figure 1:**
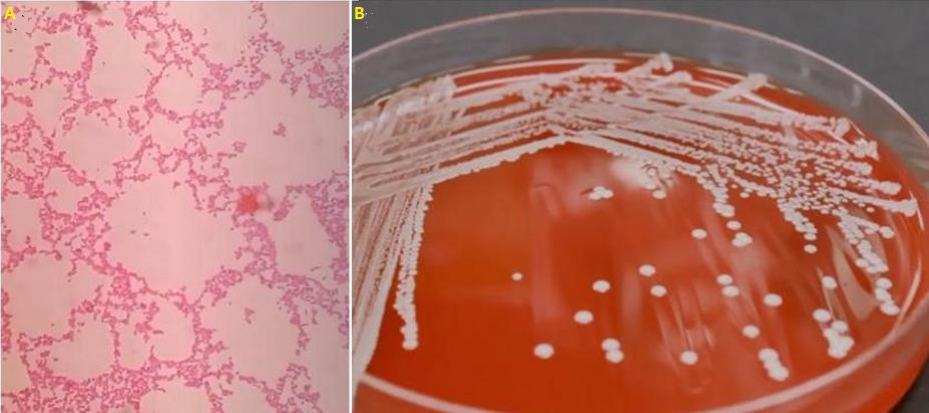
A) blood agar showing white mucoid colonies consistent with *Acinetobacter ursingii*; B) gram stain of blood culture with gram negative coccobacillary forms

**Diagnostic assessment:** antibiotic therapy was started with meropenem and vancomycin, and cultures were taken, two from peripheral blood and one from the hemodialysis-catheter tip, with isolation of *A. ursingii* on the three of them (Figure 1).

**Diagnosis:** the antibiotic susceptibility pattern is described in Table 2.

**Table 2:** antibiogram and resistance profile of *A. ursingii* isolated in hemodialysis-catheter tip culture

Antibiotic	MIC	Susceptibility
Ampicillin/sulbactam	15	Resistant
Cephalothin	92	Resistant
Cefoxitina	32	Resistant
Ceftriaxone	64	Resistant
Cefepime	35	Resistant
Meropenem	0.11	Sensitive
Imipenem	0.19	Sensitive
Amikacin	3	Sensitive
Levofloxacin	0.22	Sensitive
Fosfomycin	115	Resistant
Colistin	0.25	Sensitive

MIC: minimum inhibitory concentration

**Therapeutic interventions:** based on the results, vancomycin was suspended and antibiotic treatment was continued with meropenem.

**Follow-up and outcome of interventions:** eight days later the patient presented sudden refractory hypotension, bradycardia, cardiac arrest and death.

**Patient perspective:** the patient could not share their perspective.

**Informed consent:** written informed consent was obtained from the patient to publish this paper.

## Discussion

*Acinetobacter ursingii* is a gram-negative coccobacillus, non-motile, non-fermenter, catalase and oxidase negative; frequently found in humid environments [1], its name comes from the microbiologist and taxonomist Jan Ursing, who first described it in 1989 [2]. Until the year 2021 there has been reported only 7 cases of bacteremia due to this microorganism in adults. The main characteristics of these cases, including ours, are presented in Table 3.

**Table 3:** clinical characteristics of *Acinetobacter ursingii* infections

Case report	Age	Gender	Clinical records	Presentation	Infectious source	Antimicrobial resistance	Treatment	Days	Dialysis	Immune system	ICU	Organic dysfunction
Loubinoux *et al*. 2006	63	M	Pulmonary adenocarcinoma	Bacteremia	Central venous catheter	CAZ (ESBL)	IPM + AMK + RIF	14	No	Compromised	-	NR
Velioglu *et al*. 2016	33	F	Chronic kidney disease, chronic pyelonephritis	Peritonitis	Peritoneal dialysis catheter	-	CAZ + AMK	21	Yes	Competent	-	NR
Salzer *et al*. 2016	47	F	Intravenous drug user	Bacteremia, septic shock	None	CRO (ESBL)	MEM	10	No	Competent	-	Cardiovascular
Ducasse *et al*. 2008	47	F	Cholecystectomy, ERCP + sphincterotomy	Bacteremia, Cholangitis	None	CZO	CTX + CXM	14	No	Competent	-	NR
Chew *et al*. 2018	47	F	Acute myeloid leukemia, chemotherapy	Bacteremia	None	-	MEM	10	No	Compromised	-	NR
PR Gómez *et al*. 2006	63	F	Gastric adenocarcinoma, chemotherapy	Bacteremia	Central venous catheter	AMC	LVX	15	No	Compromised	-	NR
Holloman *et al*. 2016	27	F	Pregnancy, Hyperemesis gravidarum	Bacteremia	Peripheral venous catheter	GEN	MEM	10	No	Competent	-	NR
Endo *et al*. 2012	-	M	Chronic kidney disease, DM, SAH, oropharyngeal carcinoma	Bacteremia	Central venous catheter	MEM, FEP	CIP	14	Yes	Compromised	Yes	Cardiovascular, respiratory, renal
Arsanios *et al*. 2020	78	M	SAH, smoking history	Bacteremia	Central venous dialysis catheter	CRO (ESBL)	MEM	14	Yes	Compromised	Yes	Cardiovascular hepatic, renal, respiratory

M: male; F: female; ERCP: endoscopic retrograde cholangiopancreatography; ESBL: extended spectrum beta-lactamase; DM: diabetes mellitus; SAH: systemic arterial hypertension; CAZ: ceftazidime; CRO: ceftriazone; CZO: cefazolin; AMC: amoxicillin/clavulanic acid; GEN: gentamicin; MEM: meropenem; FEP: cefepime; IPM: imipenem; AMK: amikacin; RIF: rifampicin; CTX: cefotaxime; CXM: cefuroxime; LVX: levofloxacin; CIP: ciprofloxacin; NR: not reported

All of the reported patients had comorbidities (specially tumoral), were hospitalized, with severe disease courses and most of them were immunosuppressed [3]. Two of the cases had multi-organic dysfunction, however, only the reported by Endo *et al*. [4] presented renal dysfunction just like our case. In a retrospective study, where 456 cultures of *Acinetobacter spp* were analyzed, the patients with *A. ursingii* infections had prolonged intensive care unit (ICU) stays, were immunocompromised, underwent several invasive procedures or had multiple invasive devices, specially central venous catheters [5]. It has been demonstrated that *Acinetobacter spp* have the ability to invade skin and intravascular catheters [6,7], nonetheless, the great majority of the cases presented primary bacteremia without an identifiable source of infection or dissemination. Atas DB *et al*. reported the only case where infection was directly associated with renal replacement therapy, but the modality was intraperitoneal dialysis in a patient with chronic kidney disease presenting catheter-related peritonitis [8].

The association between acute kidney injury requiring hemodialysis and infection due to *A. ursingii* had not been described previously in the literature and its early identification might be of great clinical and epidemiological relevance. Since it is an opportunistic nosocomial microorganism with a long lasting survival on surfaces, its dissemination between patients is easy [9], increasing the risk of outbreaks in hospital environments and high circulation areas like renal units. Regarding its antimicrobial treatment, the isolations described so far are a 100% resistant to third and fourth generation cephalosporins and 80% sensitive to imipenem, levofloxacin, amikacin, gentamicin and colistin; which is consistent with the findings presented by Laurent Dortet *et al*. and Chao *et al*. [5,10], and the ones described in our patient. Nonetheless, due to the low frequency of its isolations and the limited clinical trials published so far, the full mechanisms behind its antimicrobial resistance are yet to be described.

## Conclusion

Bacteremia secondary to *A. ursingii* in hemodialysis patients is a rare cause of mortality. This would be the first reported case in the literature of a CRBSI due to this microorganism in a patient with hemodialysis. It is not possible to determine a strong association between infection and risk factors due to the rarity of this microorganism. It seems to be more frequent in severely ill patients with multiple comorbidities and invasive devices, so awareness should be taken in these kinds of patients in order to decrease the probability for *A. ursingii* infections.

## References

[1] Chew KL, Chew KL (2018). *Acinetobacter ursingii* masquerading as Gram-positive cocci. Clin Microbiol Infect.

[2] Romero-Gómez MP, Sundlov A, Sáez-Nieto JA, Alvarezc D, Peña P (2006). Bacteriemia por Acinetobacter. Enferm Infecc Microbiol Clin.

[3] Chiu CH, Lee Y-T, Wang YC, Yin T, Kuo SC, Yang Y-S (2015). A retrospective study of the incidence, clinical characteristics, identification and antimicrobial susceptibility of bacteremic isolates of Acinetobacter ursingii. BMC Infect Dis.

[4] Endo S, Sasano M, Yano H, Inomata S, Ishibashi N, Aoyagi T (2012). IMP-1-producing carbapenem-resistant *Acinetobacter ursingii* from Japan. J Antimicrob Chemother.

[5] Dortet L, Legrand P, Soussy C-J, Cattoir V (2006). Bacterial identification, clinical significance and antimicrobial susceptibilities of *Acinetobacter ursingii* and *Acinetobacter schindleri* two frequently misidentified opportunistic pathogens. J Clin Microbiol.

[6] Salzer HJF, Rolling T, Schmiedel S, Klupp E-M, Lange C, Seifert H (2016). Severe community-acquired bloodstream infection with *acinetobacter ursingii* in person who injects drugs. Emerg Infect Dis.

[7] Seifert H (1995). Acinetobacter species as a cause of catheter-related infections. Zentralbl Bakteriol.

[8] Atas DB, Velioglu A, Asicioglu E, Tigen E (2016). Peritoneal dialysis-related peritonitis with *acinetobacter ursingii*. Ther Apher Dial.

[9] Wendt C, Dietze B, Dietz E, Rüden H (1997). Survival of *Acinetobacter baumannii* on dry surfaces. J Clin Microbiol.

[10] Chao C-T, Lee S-Y, Yang W-S, Chen H-W, Fang C-C, Yen C-J (2014). Acinetobacter peritoneal dialysis peritonitis: a changing landscape over time. PLoS One.

